# Preliminary demonstration of a novel intraocular lens power calculation: the O formula

**DOI:** 10.1097/j.jcrs.0000000000000983

**Published:** 2022-06-01

**Authors:** So Goto, Naoyuki Maeda, Kazuhiko Ohnuma, Tjundewo Lawu, Ryo Kawasaki, Shizuka Koh, Kohji Nishida, Toru Noda

**Affiliations:** From the Department of Ophthalmology, Osaka University Graduate School of Medicine, Suita, Osaka, Japan (Goto, Maeda, Kawasaki, Koh, Nishida); Department of Ophthalmology, National Hospital Organization, Tokyo Medical Center, Meguro-ku, Tokyo, Japan (Goto, Noda); School of Optometry, University of California Berkeley, California (Goto); Laboratorio de Lente Verde, Sodegaura, Chiba, Japan (Ohnuma); VO, Toda, Saitama, Japan (Lawu); Department of Vision Informatics, Osaka University Graduate School of Medicine, Suita, Osaka, Japan (Kawasaki); Integrated Frontier Research for Medical Science Division, Institute for Open and Transdisciplinary Research Initiatives (OTRI), Osaka University, Suita, Osaka, Japan (Nishida).

## Abstract

To evaluate the performance of a new formula of intraocular lens (IOL) power calculation (the O formula) based on ray tracing without commonly used parameters, including ultrasound-compatible axial length, keratometry readings, and A-constant.

**Setting:**

Tokyo Medical Center, Tokyo, Japan.

**Design:**

Retrospective consecutive case series.

**Methods:**

423 eyes (423 patients) implanted with a single-piece, L-loop, acrylic IOL were enrolled. All biometric data for the O formula were obtained by anterior segment swept-source optical coherence tomography (SS-OCT) and SS-OCT–based biometer. The performance of the O formula was compared with those of the Barrett Universal II (BUII) and Kane formulas at 1 month postoperatively. Statistical analysis was applied according to a heteroscedastic test with SD of prediction errors as the main parameter for formula performance.

**Results:**

The SD of the O formula (0.426) was statistically significantly lower than that of the BUII formula (0.464, *P* = .034) but not statistically significantly different from that of the Kane formula (0.433, *P* = .601). The percentages of patients with refractive prediction errors within ±0.50 diopter (D) and ±1.00 D of the O, BUII, and Kane formulas were 75.4% and 98.6%, 77.1% and 97.9%, and 76.6% and 98.1%, respectively.

**Conclusions:**

The O formula, based on ray tracing using SS-OCT–based devices, is one of the promising approaches for IOL power calculation, although additional larger scale studies are needed. It may be used as an alternative in IOL power calculation because of its independence from commonly used parameters.

## Abstract 2

A new ray-tracing–based formula for calculating the power of intraocular lenses was developed. This formula does not rely on parameters such as ultrasound-compatible axial length, keratometry readings, and A-constant.

The accuracy of refractive outcomes after cataract surgery has been improving with the developments in surgical techniques, preoperative measurements, intraocular lenses (IOLs), and IOL power calculation formulas. Commonly used formulas for calculating IOL power are classified as vergence, artificial intelligence (AI), ray tracing, or a combination of these, and their accuracies in calculating IOL power are excellent.^1^ Although IOL power calculation theoretically lies in the domain of optical science, no formula is perfect. Even in uncomplicated cataracts, approximately 5% of cases experience prediction errors in refraction greater than 1.0 diopter (D).^2–5^

In addition to the established formulas such as the Haigis, Hoffer Q, Holladay 1, and SRK/T formulas, new formulas for IOL power calculation including the Barrett Universal II (BUII), Emmetropia Verifying Optical (EVO, unpublished), Radial Basis Function (RBF3.0), Holladay 2 with axial length (AL) adjustment, Kane, and Olsen formulas, and more, have been introduced with the aim of improving the accuracy of postoperative refraction prediction.^6–12,A–C^ All these formulas use 3 common parameters: ultrasound-compatible AL, keratometry (K) readings, and a single IOL constant that is compatible with the A-constant, which have the advantage of versatility and can be used in any facility. The AL value can be measured by A-scan immersion ultrasonography and optical biometers. K readings are available for manual or automated keratometer, corneal topographer, corneal tomographer, or AL biometer. Even for a new IOL in a particular clinic, the IOL power can be calculated using the same formula as long as the A-constant is known. While addressing the versatility of this field, inherent problems have arisen with these parameters, which may affect the postoperative refractive error.

An optical biometer can measure the AL more accurately than an A-scan ultrasound; however, the ultrasound-compatible AL calculated by the optical biometer is the distance from the anterior surface of the cornea to the internal limiting membrane, not to the retinal pigment epithelium. The retinal thickness is not measured and estimated for compatibility with ultrasound-based values.^6,13^ The algorithm that converts optical AL to ultrasound-compatible AL overestimates AL value in longer eyes and underestimates it in shorter eyes, causing hyperopic refractive errors in the long axial eye and myopic refractive errors in the short axial eye when using the vergence formula.^6,10,13^ Although optical AL is commonly measured with a composite method using an average refractive index for the entire eye, the segmented method that uses the refractive indexes of the cornea, aqueous, lens, and vitreous is able to measure AL more accurately than the composite method.^13–15^

Corneal power is calculated based on 2 anterior curvatures at the paracentral zone of the cornea in keratometry. A keratometric index of 1.3375 or 1.3315 is used to convert the anterior corneal curvature into diopters without posterior curvature data.^16^ If the Gullstrand ratio, the ratio of the anterior and posterior surfaces of the cornea curvature, is not constant (eg, in eyes after corneal refractive surgery), an error in K readings will occur.^17^

IOL manufacturers and many researchers have advised the optimization of IOL constants rather than dependence on those provided by manufacturers to achieve higher rates of accuracy. However, the necessity to optimize an IOL constant for atypical eyes remains controversial.^18^ Using the same A-constant from low to high power for 1 type of IOL would also result in refraction error due to the dependence of A-constants on the AL.^19^

Ray tracing uses physical dimensions and refractive indices of all optical media and is a promising method for calculating IOL power.^12,20–22^ Although the radii of anterior and posterior corneal surfaces and the optical AL should be used in ray tracing, some ray-tracing methods have used ultrasound-compatible AL and K readings. Because the severity of nuclear cataracts may alter the refractive index of the crystalline lens, the cataract grade should be considered in the correction of optical AL.^23^ Moreover, information on IOLs, such as the refractive index and the radius of curvatures for all powers of IOL, remains uncertain.

The recent evolution of optical biometry with swept-source optical coherence tomography (SS-OCT) technology and anterior segment SS-OCT has substantially improved the measurement accuracy.^24,25^ We devised a new ray-tracing–based IOL power calculation formula, termed the O formula, using anterior segment SS-OCT and SS-OCT–based biometer. The O formula adopts 4 new concepts: (1) the AL, which is the distance from the corneal surface to the photoreceptors, measured by the segmented method and adjusted depending on the grade of cataract; (2) prediction of IOL depth (corneal endothelium to IOL surface) using anterior segment parameters with the OCT image; (3) the actual shape and refractive index for each IOL power; and (4) optical corneal power using anterior and posterior curvatures and corneal thickness based on the Snell law.

The aim of this preliminary study was to evaluate the accuracy of the O formula compared with that of the BUII and Kane formulas in a series of patients undergoing cataract surgery

## METHODS

### Selection Criteria

This study was designed as a retrospective consecutive case series and was approved by the Institutional Review Board of the National Hospital Organization, Tokyo Medical Center (R18-161). Written informed consent was obtained from all patients, and the study was conducted in accordance with the tenets of the Declaration of Helsinki. All patients were recruited from the National Hospital Organization, Tokyo Medical Center, between December 2015 and June 2020. Only eyes undergoing their first cataract surgery performed by 1 experienced surgeon (T.N.) were included. The exclusion criteria for the study were as follows: corrected distance visual acuity after cataract surgery less than or equal to 20/40; preoperative or postoperative astigmatism greater than 4.0 D; a history of ocular surgery and/or ocular trauma, the presence of a significant ocular comorbidity (eg, ocular surface diseases, keratoconus, pterygium, or pseudoexfoliation syndrome); unreliable or undetectable preoperative biometry measurements; or a history of intraoperative or postoperative complications.

All patients underwent routine preoperative and 1-month postoperative ophthalmic examinations, including a corrected distance visual acuity measurement using a Landolt C chart at 5 meters, slitlamp examination, keratometry, intraocular pressure measurement, and fundoscopy. The postoperative refraction measurements were performed by experienced certified orthoptists. SS-OCT–based biometer (OA-2000; Tomey Corp.) and anterior segment SS-OCT (CASIA2) were performed preoperatively and 1 month postoperatively. All examinations were performed by experienced ophthalmic technicians blinded to the purpose of the study.

The surgical technique comprised a 2.2 mm temporal clear corneal sutureless incision and phacoemulsification. A single-piece, L-loop, acrylic IOL (AcrySof Toric IOL, SN6A T3-T6; Alcon Laboratories, Inc.) was implanted into the bag after a continuous curvilinear capsulorrhexis in all cases.

### Definitions of Anterior Segment Parameters

**Figure 1.**
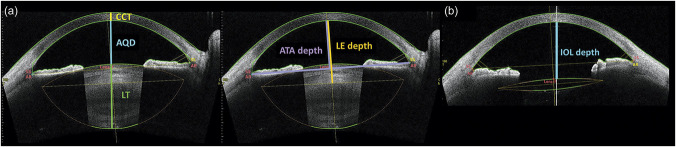
A horizontal cross-sectional image by anterior segment swept-source optical coherence tomography before (*a*) and after (*b*) cataract surgery. AQD = aqueous depth; AR = angle recess; ATA depth = angle-to-angle depth; CCT = central corneal thickness; LE depth = lens equator depth; Lens-f = lens fornix; LT = crystalline lens thickness; SS = scleral spur

**Figure 2.**
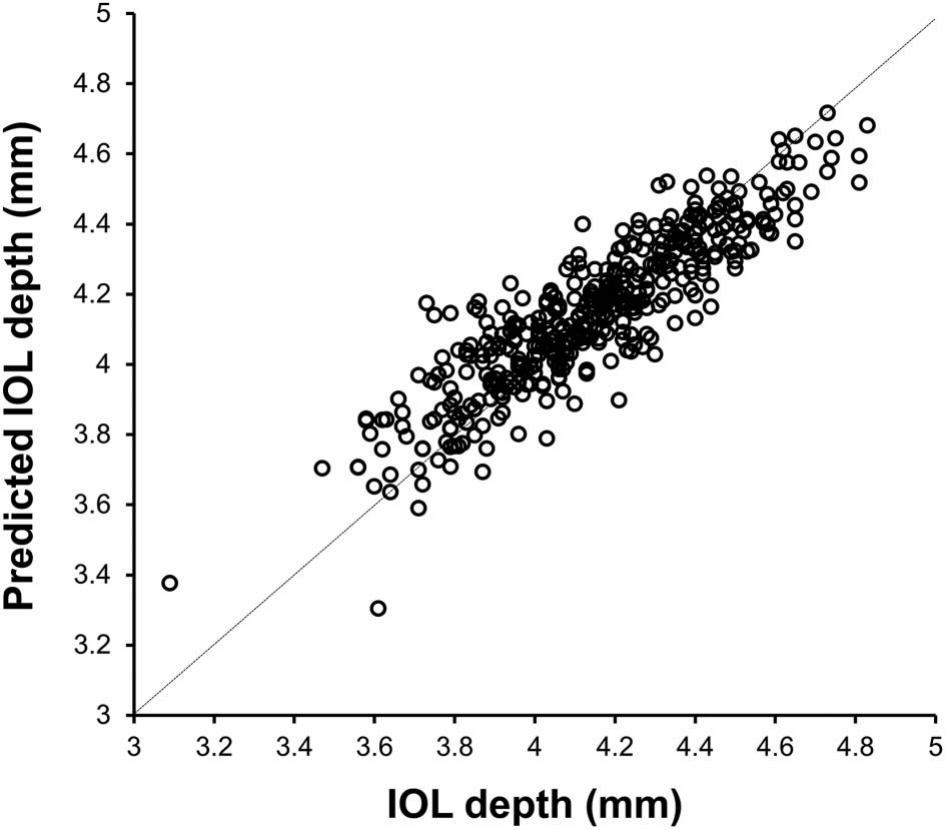
Scatterplot of the correlation between postoperative IOL depth and predicted IOL depth (n = 423).

Anterior segment parameters measured using SS-OCT were defined as follows (Figure 1). Cross-sectional images were obtained by OCT with a horizontal (180 degrees) alignment and were centered on the corneal vertex, which was defined as the cross point of the vertex normal and anterior corneal surfaces. Central corneal thickness (CCT) was defined as the distance between the anterior and posterior corneal surfaces. The distance between the posterior corneal surface and anterior lens surface was defined as the preoperative aqueous depth (AQD). The definition of crystalline lens thickness (LT) was the distance between the anterior and posterior lens surfaces. CCT, AQD, and LT were measured along the vertex normal.^13^ Angle-to-angle (ATA) depth was defined as the perpendicular distance between the posterior corneal surface and a line drawn between the anterior chamber angle recesses on the nasal and temporal sides of the horizontal OCT scans.^25^ The lens equator (LE) depth was defined as the perpendicular distance from the posterior surface of the cornea to the crystalline lens equator line.^26^ The crystalline lens equator line was determined by 2 meridians of the lens equator, which was estimated based on an imaginary line connecting the anterior and posterior lens surfaces. The postoperative IOL depth was measured as the distance between the posterior corneal surface and the anterior IOL surface along the vertex normal (Figure 2). All parameters were automatically calculated using a customized program on the anterior segment SS-OCT.

### Calculation of AL and Predicted IOL Depth

The segmented AL was measured as the sum of the thicknesses of the cornea, aqueous, lens, and vitreous.^13–15^ The refractive indices of the cornea, aqueous, lens, and vitreous were set to 1.376, 1.336, 1.410, and 1.338, respectively.^27,28^ The segmented AL from the cornea to the photoreceptors (external limiting membrane [ELM]) was calculated using the following formula (1):

(1) Segmented AL =CCT + AQD + LT × 1.410/1.426 + (1.3496 × (0.9573×AL+ 1.3304) + 1.376 × CCT + 1.336 × AQD +1.410 × LT)/1.338 + EL

where AL indicates the axial length, CCT the central corneal thickness, AQD the preoperative aqueous depth, LT the crystalline lens thickness, and EL the external limiting membrane thickness (the distance between the ELM and retinal pigment epithelium). All variables were in millimeters and obtained from anterior segment SS-OCT except for AL, which was determined from the SS-OCT–based biometer. If the cataract was more than a grade 3 in the LOCS III, a different refractive index (1.450) was used for the crystalline lens.^28,29^

The predicted IOL depth was calculated using the regression formula (2) modified from the original one.^25^ LE depth and LT, which can be measured by using CASIA2, were adopted as a new parameter. The regression formula (2) was previously developed based on 315 eyes that had undergone cataract surgery performed by the same surgeon (T.N.); however, those eyes were independent to the patients of this study. Biometric data of this population were AL (24.09 ± 1.55 mm), K reading (44.11 ± 1.51 D), anterior chamber depth (3.08 ± 0.39 mm), and LT (4.70 ± 0.39 mm).

(2) IOL depth = (a) × segmented AL (b) × (LT/2 + AQD) (c) × ATA depth (d) × LE -depth (e)

where AL is the axial length, LT the crystalline lens thickness, AQD the preoperative aqueous depth, ATA depth the angle-to-angle depth, and LE depth the lens equator depth. All variables were in millimeters and obtained from AS-SS-OCT, except segmented AL. (a), (b), (c), (d), and (e) are arbitrary coefficients.

### IOL Power Calculation

Based on the corneal biometry, predicted IOL depth, segmented AL, and IOL design, the refractive errors for all available dioptric powers were calculated using previously published ray-tracing methods (see the Appendix, http://links.lww.com/JRS/A607).

### Data Collection and Patient Examinations

The BUII (v.1.05) and Kane formulas were calculated through their respective websites with the IOL selection of SN6Tx (A-constants: 119.26 and 119.28, respectively).^11,30^ The prediction error was obtained as the postoperative manifest refraction minus the predicted refractive values calculated by 3 formulas using the implanted IOL power. The mean numerical prediction error, SD of prediction error, mean absolute prediction error (MAE), and median absolute prediction error (MedAE) were calculated for each formula. The percentages of eyes with a prediction error of ±0.25 D, ±0.50 D, and ±1.00 D or less were calculated for each formula. Eyes were separated into subgroups based on the AL for subgroup analysis as follows: short (≤22 mm), medium (>22 to <26 mm), and long (≥26 mm).

### Statistical Analysis

Statistical analyses were performed with JMP Pro v. 14.0.0 (SAS Institute, Inc.). The heteroscedastic test was performed to compare the SDs obtained by the 3 formulas.^31^ The mean numerical prediction error was adjusted to zero for each formula to eliminate the systematic error from the chosen lens constant. Dr. Barrett and Dr. Kane performed the optimization and data analyses of the BUII and Kane formulas, respectively. The Friedman test was used to compare differences in MedAE among formulas. The Cochran Q test was performed to compare the number of eyes within ±0.25 D, ±0.5 D, and ±1.0 D of refractive prediction errors among the IOL formulas. The sample size was calculated to detect a difference in the median absolute error of 0.25 D between the 2 groups, with a significance level of 5% and a statistical power of 80%, assuming SD to 0.5 D; 128 eyes were thus required. *P* values less than 0.05 were considered statistically significant.

## RESULTS

A total of 423 eyes (423 patients) were enrolled; Table 1 summarizes the demographic data and ocular dimensions including the CCT, AQD, and LT.

**Table 1.** Demographic and ocular characteristics (n = 423)

Characteristic	Mean ± SD (range)
Age (y)	76.5 ± 8.3 (44, 96)
F, n (%)	262 (62)
IOL power (D)	20.2 ± 3.9 (6, 28.5)
NCG (LOCS III)	2.2 ± 0.4 (1.0, 5.0)
SS-OCT–based OB	
AL (mm)	24.31 ± 1.54 (21.33, 29.82)
CP (D)	44.07 ± 1.52 (39.49, 48.90)
CT (μm)	532 ± 30 (442, 626)
ACD (mm)	3.12 ± 0.38 (2.19, 4.23)
LT (mm)	4.67 ± 0.39 (3.55, 5.78)
AS SS-OCT	
CCT (μm)	545 ± 32 (449, 642)
AD (mm)	2.69 ± 0.41 (1.43, 3.74)
LT (mm)	4.68 ± 0.39 (3.57, 5.86)
ATA depth (mm)	3.25 ± 0.21 (2.45, 3.87)
LE depth (mm)	4.14 ± 0.32 (2.53, 4.94)
IOL depth (mm)	4.14 ± 0.27 (3.09, 4.83)
Anterior CC (steep) (mm)	7.61 ± 0.28 (6.84, 8.47)
Anterior CC (flat) (mm)	7.72 ± 0.26 (7.01, 8.71)
Anterior CE	0.52 ± 0.19 (–0.40, 1.05)
Posterior CC (steep) (mm)	6.26 ± 0.28 (5.40, 7.19)
Posterior CC (flat) (mm)	6.52 ± 0.26 (5.86, 7.37)
Posterior CC	0.67 ± 0.14 (–0.12, 1.04)

ACD = anterior chamber depth, measured from epithelium to lens; AD = aqueous depth; AD = aqueous depth; AL = axial length; ATA = angle to angle; CC = corneal curvature; CE = corneal eccentricity; CCT = central corneal thickness; CE = corneal eccentricity; CP = corneal power; CT = corneal thickness; LE = lens equator; LOCS = Lens Opacities Classification System; LT = lens thickness; NCG = nuclear cataract grade; OB = optical biometer

The mean and SD of the postoperative IOL depth was 4.14 ± 0.27 mm (range: 3.09 to 4.83 mm). The predicted IOL depth was 4.15 ± 0.22 mm (range: 3.30 to 4.72 mm). The *R*^2^ value between the predicted and postoperative IOL depth was 0.79 (*P* < .0001). Figure 2 shows the scatterplot between the predicted IOL depth and measured values. The MAE in IOL depth prediction was 0.09 ± 0.08 mm.

**Figure 3.**
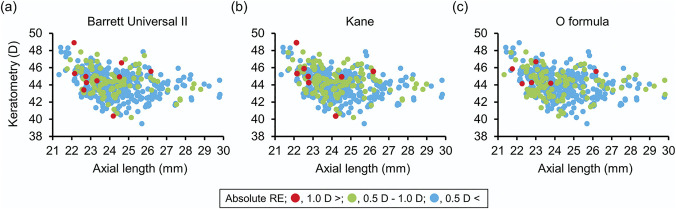
Scatterplot with 3 classes of absolute refractive error (RE) calculated using the Barrett Universal II formula (*a*), Kane formula (*b*), and O formula (*c*) in relation to axial length and the average keratometry value obtained by swept-source optical coherence tomography–based biometer. Absolute REs were color-coded into 3 classes: red dot, more than 1.0 D; green dot, 0.5 D or more and 1.0 D or less; and blue dot, less than 0.5 D.

The results of each formula are summarized in Table 2. As the result of the heteroscedastic test, the SD of the O formula (0.426) was statistically significantly lower than that of the BUII formula (0.464, *P* = .037) but not statistically significantly different from the Kane formula (0.433, *P* = .607). The SD of the Kane formula was statistically significantly lower than that of the BUII formula (*P* < .001). Figure 3 shows the color-coded scatterplots of absolute refractive prediction errors by each formula with the AL and the average K reading.

**Table 2.** Overall outcomes for the Barrett Universal II, Kane, and O formulas

Formula	Mean ± SD	Range	MAE ± SD	MedAE	Eyes within range indicated (%)		
					±0.25 D	±0.50 D	±1.00 D
Without adjusting mean RPE to zero							
Barrett Universal II	0.083 ± 0.464	−1.00, 1.59	0.373 ± 0.288	0.320	40.0	72.8	96.9
Kane	−0.034 ± 0.433	−1.27, 1.30	0.349 ± 0.259	0.295	41.4	76.6	97.9
O	0.093 ± 0.426	−0.97, 1.23	0.349 ± 0.260	0.303	43.5	75.2	97.9
After adjusting mean RPE to zero							
Barrett Universal II	0.000 ± 0.439	−1.09, 1.45	0.351 ± 0.264	0.290	43.5	77.1	97.9
Kane	0.000 ± 0.434	−1.23, 1.35	0.347 ± 0.261	0.295	41.8	76.6	98.1
O	0.000 ± 0.429	−1.09, 1.16	0.343 ± 0.256	0.290	43.0	75.4	98.6

MAE = mean absolute prediction error; MedAE = median absolute prediction error; RPE = refractive prediction error

The subgroup analysis is summarized in Table 3. There was no statistically significant difference among the 3 formulas in the short AL eyes group (10 eyes). In the subgroup of the medium AL eyes (352 eyes), the SD of the O formula was statistically significantly better than that of the BUII formula (*P* = .008), whereas no statistically significant difference was found between the Kane and O formulas (*P* = .21). There was no statistically significant difference among the 3 formulas in the subgroup of the long AL eyes (61 eyes).

**Table 3.** SD, MedAE, and MAE for each formula by axial length

Formula	SD	MedAE	MAE
Short (n = 10)			
Barrett Universal II	0.398	0.273	0.383
Kane	0.363	0.340	0.361
O	0.570	0.392	0.408
Medium (n = 352)			
Barrett Universal II	0.467	0.315	0.353
Kane	0.437	0.305	0.348
O	0.416	0.275	0.339
Long (n = 61)			
Barrett Universal II	0.427	0.344	0.315
Kane	0.412	0.335	0.305
O	0.454	0.356	0.333

MAE = mean absolute prediction error; ME = mean prediction error; MedAE = median absolute prediction error

Short (≤22 mm), medium (>22 to <26 mm), and long (≥26 mm)

## DISCUSSION

The results of this preliminary study indicate that the new IOL formula based on a ray-tracing method without ultrasound-compatible AL, keratometry readings, and A-constant performs as well as the currently available and highly accurate theoretical IOL formulas that adopt these commonly used parameters in participants with uncomplicated cataracts. The O formula produced the most accurate outcomes regarding the lowest SD and MAE of refractive prediction. The results of this study are most similar to those observed in the study conducted by Darcy et al., who reported that 70.7% (the BUII formula) and 72.0% (the Kane formula) of 10 930 eyes achieved a prediction error within 0.50 D.^5^

The O formula is based on ray tracing, which requires physical dimensions and refractive indices of all optical media. For pseudophakic eyes, accurate information on the cornea and IOL regarding the shape of their surfaces and distance between each interface is required. In such cases, ultrasound-compatible AL and K readings may be the cause of prediction errors as these parameters are optimized for vergence formulas. Furthermore, the A-constant cannot be directly used in ray-tracing calculations. Therefore, the O formula uses differently defined AL and corneal parameters, prediction of IOL position, and actual data of physical dimensions and refractive index of IOL for all the available power instead of legacy parameters, such as the conventional ultrasound-compatible AL, K readings, and A-constant.

A theoretical reconsideration of the precise definition of AL for ray-tracing formulas is needed. Although the existing IOL power calculation formulas have used the displayed AL, which is compatible with immersion-ultrasound AL (the distance from the corneal surface to the internal limiting membrane), the distance between the corneal surface and photoreceptors is essential for calculating the IOL power in ray tracing. We speculate that photons should be focused on the base of the inner segment (ie, the ELM) to increase efficiency, as the segmented structure of the photoreceptors operates similar to an optic fiber. Indeed, the Stiles-Crawford effect can be explained by this hypothesis.^32,33^ In the O formula, the geometrical path length between the corneal surface and the base of the inner segment is used as the optical true AL. In addition, the O formula adopts the segmented AL (also called the sum-of-segments AL) obtained by SS-OCT–based devices.

Regarding the refractive index of a crystalline lens, cataract severity should be considered in the calculation of the geometric lens length because it may change the refractive index of the crystalline lens depending on the cataract grade.^34^ A nuclear cataract causes a significant myopic shift, which is partly explained by the increasing refractive index of the lens.^35^ Specifically, if the optical path length of the lens is 6.0 mm, the thickness of the lens is calculated to be 4.26 mm when the refractive index is 1.41 (normal cataract); however, it is calculated to be 4.14 mm when the refractive index is 1.45 (high-grade nuclear cataract), resulting in a difference of 0.12 mm. Therefore, the O formula uses a different refractive index for the lens in cases of high-grade nuclear cataracts.

Regardng corneal power, the corneal axial power is most often used based on the 2 radii of the anterior corneal surface at the paracentral zone using a Placido-based keratometer; these radii are converted to K readings, not with the true refractive index of the cornea stroma (1.376) but with the keratometric index (1.3375 or 1.3315). The keratometric index is used based on the assumption that the Gullstrand ratio of the anterior surface curvature to the posterior surface curvature is constant.^16^ K readings using the keratometric index with a normal cornea are higher than the corneal optical power calculated with ray tracing by approximately 0.5 to 0.9 D.^36^ K readings are suitable for the existing vergence IOL power calculation formulas but would not be ideal for IOL power calculation with ray tracing.

As opposed to the virtual effective lens position (ELP) of the vergence formulas, the accurate prediction of the postoperative IOL depth is one of the most important factors in ray-tracing IOL power calculation. Previously, we measured the IOL depth using SS-OCT and compared it with the values of the predicted ELP according to the Haigis and SRK/T formulas. The results revealed that the Haigis and SRK/T formulas overestimated IOL depth relative to actual IOL depth values.^25^ The SS-OCT achieved a more accurate prediction of the postoperative IOL depth compared with the ELPs of the Haigis and SRK/T formulas.^25,26^ The simple replacement of the predicted IOL depth with SS-OCT inside previous IOL formulas will not improve the refractive results because these formulas are optimized for the overestimated ELP to achieve the smallest refractive prediction error. In this study, the mean prediction error of IOL depth was within 0.1 mm. This prediction accuracy of IOL depth was equivalent or better than that found in previous studies.^25,26,37,38^

This study has several limitations. The first is its retrospective single-center design and the relatively small patient population that included only uncomplicated cataracts within a single ethnic group. Although these preliminary results validated the accuracy and predictability of the new IOL formula compared with only 2 other formulas, a prospective multicenter study with a larger group of patients using multiple formulas is required to confirm our findings.^5,39^ The second limitation is that the O formula requires the physical information on the optical design of each IOL. The O formula must be developed from scratch for each IOL on an individual basis, which is time-consuming. However, once the formula is developed for a particular IOL, we believe that the accuracy will improve considerably. This proprietary information must be provided by the manufacturer, and an algorithm for predicting IOL depth should be developed for each IOL. Although the IOL information for each power was provided by the manufacturer in this study, ascertaining these proprietary details has proven to be a major obstacle to developing theoretical IOL power formulas for various IOLs. Subsequently, a customized program on the anterior segment SS-OCT device is required to calculate some of the anterior segment parameters that are needed to predict IOL depth. All parameters are required to be checked for their segmentation. When automated segmentation is incorrect, manual correction is required. In the near future, we expect that technologies such as AI and machine learning will reduce the need for manual correction. Third, in this study, the prediction of IOL depth was dependent on a specific anterior segment SS-OCT device; this reliance on a single device could affect the reproducibility of the study, and the reproducibility of the measurement across different devices should thus be evaluated. Finally, it should be noted that the O formula is not a satisfactory result for long and short eyes. As only patients with normal corneas were included in this study, further investigations are needed to elucidate whether the corneal optical power used in this study is effective for eyes with abnormal corneal shapes (eg, laser in-situ keratomileusis, radial keratotomy, corneal transplantation, and corneal diseases).

At present, a subset of patients with uncomplicated cataracts still presents with refractive errors greater than 1.0 D. The commonly used parameters, including ultrasound-compatible AL, corneal keratometry readings, and A-constant, which many formulas depend on, are useful and convenient, but they may cause inherent refractive errors in some cases. We believe that the O formula is a theoretically robust approach for accurate IOL power calculation using ray tracing and may be useful as an independent method to avoid the refractive errors caused by existing IOL power calculation formulas with the commonly used parameters.

**WHAT WAS KNOWN**

- Advances in surgical techniques, preoperative measurements, IOLs, and IOL power calculation formulas have improved the accuracy of refractive outcomes after cataract surgery.
- However, the established IOL power calculation formulas with commonly used parameters, including ultrasound-compatible axial length, corneal keratometry readings, and A-constant, still generate postoperative refractive errors more than 1.0 D in a percentage of the patient population.

**WHAT THIS PAPER ADDS**

- The O formula may be useful as an independent method to avoid the refractive errors caused by existing formulas with commonly used parameters, although additional larger scale studies are needed.
